# Natural Macromolecules as Building Blocks for Microcapsule Formation in Drug Delivery

**DOI:** 10.3390/pharmaceutics18070839

**Published:** 2026-07-09

**Authors:** Isidora Lajevec, Nebojša Pavlović, Dejan Ćirin, Veljko Krstonošić

**Affiliations:** Department of Pharmacy, Faculty of Medicine, University of Novi Sad, Hajduk Veljkova 3, 2100 Novi Sad, Serbia; isidora.lajevec@mf.uns.ac.rs (I.L.); dejan.cirin@mf.uns.ac.rs (D.Ć.); veljko.krstonosic@mf.uns.ac.rs (V.K.)

**Keywords:** microcapsules, methods of microencapsulation, drug delivery, polymers for microencapsulation

## Abstract

**Background/Objectives**: Microcapsules are particles 1–1000 µm in size, with a core containing the active substance (in liquid, solid, or gaseous state) and a shell typically composed of natural, synthetic, or semi-synthetic polymers. Although natural polymer-based microcapsules have applications in food, cosmetics, and other industries, this review primarily focuses on their role in pharmaceutical drug delivery. In recent years, natural macromolecules have gained increasing attention as coating materials due to their biocompatibility, biodegradability, low toxicity, mucoadhesive properties, and ability to enable controlled and targeted drug release. Based on previous research, this review provides an overview of microcapsules, the most common microencapsulation methods, natural polymers used as wall materials, and their pharmaceutical applications across different routes of administration. **Results**: By encapsulating active ingredients, microcapsules enhance their bioavailability, prolong their release, protect them, enable targeted delivery, and mask unpleasant tastes and odors. Among the most commonly used microencapsulation techniques are physical methods (spray drying, spray cooling, solvent evaporation, spray coating, and freeze drying) and physicochemical methods (coacervation). Natural polymers, particularly polysaccharides and proteins, have been successfully used in oral, topical, transdermal, pulmonary, and colon-targeted drug delivery systems, as well as for the stabilization and delivery of peptides, proteins, probiotics, and vaccines. **Conclusions**: Proper selection of microencapsulation technique depends on the properties of the polymer and the core material. Natural polymers represent versatile pharmaceutical excipients owing to their biocompatibility, biodegradability, safety, mucoadhesive behavior, and ability to provide controlled and targeted drug delivery. Their successful application with a wide range of therapeutic agents and administration routes highlights their considerable potential for the development of advanced drug delivery systems.

## 1. Introduction

In recent years, the growth of nano- and microtechnology has become significant across various scientific fields, particularly in pharmaceutical sciences and drug delivery research. The focus is on modernizing production, developing innovative therapeutic products, improving their quality, safety, and efficacy, and ensuring their targeted delivery. Drug delivery systems enable controlled release and targeted delivery of drugs to specific tissues or cells in the human body; they can improve drug stability, absorption, and therapeutic index, minimize toxicity, and reduce the need for frequent dosing of drugs [1]. One of these systems is microcapsules.

Microcapsules are microscopic particulate systems in which the active substance forms the core, enclosed within a surrounding wall material that is different from the core [2]. Microcapsule size generally ranges from 1–1000 µm [3,4]. They can be spherical, multi-layered, with one or more cores, irregularly shaped, and in the form of a matrix with microspheres [5,6]. The core material can be a solid, liquid, or gas [7]. The most commonly used wall materials are polymers, which may be natural (such as alginate, gelatin, gum arabic, agarose, or chitosan), semi-synthetic (such as carboxymethyl cellulose), or synthetic (such as polylactide, polyvinyl alcohol, or polyamide). The coating material should be mechanically stable, chemically inert, impermeable, and capable of providing controlled release of the substance at a specific site, and should not impart its own taste to the final product [5,8]. Natural polymers have benefits due to their biodegradability, biocompatibility, non-toxicity, and low production cost.

Although microcapsules are used in food, cosmetic, textile, and chemical industries, their pharmaceutical applications have attracted particular attention due to their ability to improve drug stability, bioavailability, targeted delivery, and therapeutic outcomes. They can be used to encapsulate therapeutic agents, enzymes, hormones, vitamins, essential oils, probiotic bacteria, plant extracts, colorants, and various other substances. They can be transported through various biological barriers, including the bloodstream, the skin, mucosal surfaces, and the gastrointestinal tract. In addition to traditional drug delivery systems, polymer-based microparticulate systems are being developed to release drugs in a controlled manner over time [9]. This enables targeted and controlled release of active substances to specific tissues. Encapsulating active pharmaceutical ingredients (APIs) increases their bioavailability and stability, protects them from external influences, masks unpleasant tastes and odors, prevents evaporation of volatile compounds and drug incompatibilities, and increases retention of active substances on the skin and mucous membranes [10,11,12]. Enclosing solid, liquid, or gas particles within a secondary wall material protects the active compounds from light, oxidation, pH change, heat, moisture, and gastrointestinal and enzymatic degradation, preserving their stability and efficacy. Microcapsules should be designed to maintain size uniformity, retain their active components until delivery to the target site, break down fully after use, and to be cost-effective [13].

There are various methods for microencapsulation, and their selection is based on the physicochemical properties of the core material and the composition of the wall material [14]. Traditional microencapsulation methods, such as spray drying and simple coacervation, are being replaced by complex coacervation, solvent evaporation, and extrusion methods that allow greater efficiency and wider application [3].

This topic is important because existing conventional formulations often have limitations in terms of stability, bioavailability, and safety. The development of new drug delivery systems is essential for enhancing current therapies for various diseases, as well as for developing systems that will improve the three key standards vital for any drug: efficacy, quality, and safety. Microcapsules for targeted drug delivery allow medications to reach specific molecular targets in cells and tissues, enhancing therapeutic selectivity and minimizing damage to surrounding areas. Natural materials have attracted much attention in recent years due to their biocompatibility, biodegradability, and lower toxicity than synthetic materials, especially in the context of growing concerns about the accumulation of microplastics in the environment. There is a need for a systematic review that will compare different natural polymers, microencapsulation methods, and their pharmaceutical applications. In this regard, this article aims to review current trends that can help researchers select appropriate polymers and microcapsule fabrication technologies, as well as identify the advantages, limitations, and future directions in the research of these systems. Based on previous research, this review provides an overview of microcapsules, the most common microencapsulation methods, natural polymers used as wall materials, and their applications in different industries, with special emphasis on pharmaceutical drug delivery.

## 2. Fundamental Advantages of Microencapsulation

Green et al. (1956) prepared the first gelatin microcapsules, followed by the first commercial application of this technology in the production of carbonless copy paper, where the microcapsules contained dye that was released under pressure, eliminating the need for indigo paper [15]. Based on this study, further research is being carried out in the field of microencapsulation, which will spread to various industries over the next decades. Today, microcapsules play a significant role in pharmaceutical sciences and drug delivery, while also finding applications in cosmetics, food, and other industries. Their ability to protect active ingredients and enable controlled and targeted release has made them valuable carriers for a wide range of therapeutic agents.

There are many reasons for encapsulating active substances:

- Enhancing the bioavailability of the active compound

Microencapsulation enhances stability and bioavailability and enables controlled release of the drugs, ensuring maximum therapeutic effect while minimizing dosage frequency.

- Prolonged and sustained release of the core materialMicroencapsulation of the drug effectively slows down and prolongs its release, leading to enhanced retention at the site of action. This process improves patient compliance and reduces the frequency of drug use.

- Protection

Microencapsulation can protect a drug from environmental conditions such as UV radiation, oxygen, moisture, dehydration, temperature, and pH, as well as from gastrointestinal conditions and enzymatic degradation [2,3,4,5,6,13,16]. In this way, the shelf life of drugs that are sensitive to these factors can be extended. Also, by microencapsulating the drug or food components, we can achieve controlled release by preventing the premature release of the active compound. For example, in the study with encapsulated analgesic drug ropivacaine, the results indicate that microparticles formulated with an optimal polymer composition exhibit enhanced release behavior [17]. The second example is that enteric coatings protect the drug from the acidic environment of the stomach, ensuring that it remains intact in its encapsulated form until it reaches regions of the gastrointestinal tract with a higher pH.

- Targeted delivery of the drug

Microcapsules can be used as carriers for active ingredients of the drug, enzymes, vitamins, hormones, essential oils, plant extracts, and other substances. That makes them potential systems for the targeted delivery of substances to the target site in the body without affecting surrounding healthy tissues [18]. In an in vitro and in vivo study of encapsulated budesonide for the treatment of ulcerative colitis in rats, using dextran as the wall material, targeted release into the colon and a better therapeutic effect of encapsulated budesonide were achieved [19].

- Taste and odor masking

Microencapsulation helps improve patient adherence by masking undesirable tastes and odors of medications or food components. Selecting the right polymer for this purpose is challenging, as the polymer must remain insoluble in saliva at a pH of 6.8 to prevent drug release in the oral cavity, while enabling drug release under gastric conditions at a pH of 1.2 [20]. In one study, chlorpheniramine maleate was encapsulated using chitosan and alginate as the wall materials to reduce its bitter taste, and the results showed an acceptable taste [21].

- Conversion of liquid components into a powdered form

Microencapsulation transforms liquid medications into powders with good flow properties, making them easier to handle and process [4].

- Reduction in evaporation of volatile substances

Microencapsulation can reduce the evaporation of volatile compounds and improve the stability of sensitive active ingredients. In addition to pharmaceutical applications, microencapsulation technology is widely used in the cosmetics and household product industry in order to make products such as creams, shampoos, shower and bath gels, soaps, lotions, sunscreens, tanning creams, makeup, toothpastes, surface washing liquids, and fragrances [22,23]. These systems act as carriers for various ingredients in cosmetic products, such as therapeutic agents, vitamins, UV filters, antioxidants, moisturizers, pigments, essential oils, plant extracts, and other substances in order to achieve their controlled and targeted release, protect them from external factors, and reduce the potential toxicity of certain active ingredients to the skin. To make the products more attractive to customers, the unpleasant odors of the active substances and the visual and tactile appearance of the product itself are masked and improved by this technology. Also, enhanced absorption at the skin surface and increased drug penetration are achieved through microencapsulation [18]. Some examples of encapsulated active ingredients investigated for cosmetic applications include vitamin E [24], ascorbic acid [25], retinyl palmitate [26], nicotinamide [25], rosmarinic acid [27], UV filters like octyl salicylate [28], benzoyl peroxide, and tretinoin [29].

## 3. Techniques of Microencapsulation

There are different techniques of microencapsulation used in pharmaceutical sciences to prepare drug delivery systems capable of improving drug stability, bioavailability, controlled release, and site-specific delivery. Their purpose is to coat an active ingredient (core material) with a shell of coating material (wall material). The choice of the most appropriate technique depends on the core and wall material properties, required particle size, microcapsule applications, the intended release characteristics, encapsulation efficiency, large-scale production, and manufacturing costs [3,23]. The classification of microencapsulation techniques, which are divided into three categories: physical, chemical, and physicochemical, is shown in Table 1 [3,30,31]. In this review article, some of these methods, which are used in microencapsulation, will be described.

### 3.1. Spray Drying

**Figure 1:**
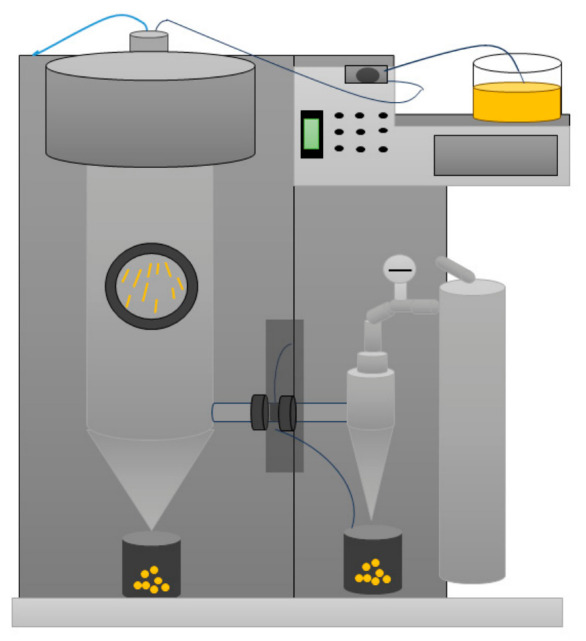
Spray drying.

Spray drying (Figure 1) is a technique in which a liquid solution, emulsion, or suspension of the core material and the wall material is introduced and dispersed into tiny droplets in a chamber with a stream of heated gas, usually air, using an atomizer. After breaking down the liquid into fine droplets, and solvent evaporation, solid particles are made. Appropriate particle characteristics, including size and shape, are achieved through processing parameters such as the composition of the mixture, atomization settings, and the temperature applied during drying [3].

Numbered lists can be added as follows:

1. The liquid is pumped through the sprayer (atomizer), where liquid drops are formed.
2. Contact between the resulting small droplets and heated gas (air, or less commonly, nitrogen), evaporation of the solvent from their surface, and formation of solid particles.
3. Collection and separation of solid, dried particles from the gas stream [3,32,33].

The liquid feed is injected through a spray nozzle into the drying chamber. The nozzle is a part of the atomizer. The atomizer breaks the initial solution, emulsion, and suspension into small liquid droplets. This stage enhances the surface area of the droplets and improves heat and mass transfer, facilitating solvent evaporation. The size of the produced droplets is determined by the spray nozzle, typically ranging from 20 µm to 185 µm [34].

When gas comes into contact with liquid droplets, the solvent evaporates, resulting in the formation of solid particles. Liquid droplets can pass through the chamber in the same direction as the heated air (co-current flow) or in the opposite direction (counter-current flow) [35]. In the co-current drying process, the inlet gas temperature usually varies from 150 to 220 °C, while the outlet gas temperature is between 50 and 80 °C [36]. Actual particle temperature is lower due to rapid evaporation and lower outlet gas temperature, which explains why the co-current drying can still be applied for heat-sensitive materials under appropriate conditions. Therefore, spray drying can be applied to certain heat-sensitive compounds only when formulation and process parameters are carefully optimized to preserve their stability and biological activity.

The process of drying and the formation of solid particles involves three phases:

1. The initial phase after contact between the liquid droplets and air, during which the drying rate increases.
2. The second phase is characterized by a constant drying rate.
3. The final phase is characterized by a decrease in the drying rate.

The temperature of the liquid droplets increases as they interact with the heated air. During the constant drying rate period, an equilibrium forms between the heat provided by the heated air stream and the heat removed through solvent evaporation, causing the liquid droplets to remain at a lower temperature than the surrounding heated air. The collection of solid particles is achieved using a cyclone separator, bag filters, and an electrostatic precipitator [37]. The most commonly used is the cyclone separator [38].

Spray drying is a frequently used method for microencapsulation because it offers several advantages, including scalability, simplicity, reproducibility, cost-effectiveness, use of different wall and core materials (even heat-sensitive compounds), fast processing, reduced energy usage, precise control over the size and shape of the dried particles, particle uniformity, and improved water solubility and bioavailability of drugs, enabling preparation of inhalation formulations. For this reason, spray drying is among the most widely used techniques for the preparation of oral and pulmonary drug delivery systems [3,5,33,39,40,41].

Although this technique has some advantages, it also has some disadvantages associated with the use of higher operating temperatures compared to other techniques, which limits its application for the effective encapsulation of volatile and thermolabile compounds [41,42,43]. Another limitation is the choice of coating, which should be water-soluble [44]. The yield is also lower due to product loss from deposition on the walls of the drying chamber [33,37]. The equipment is large in size and large amounts of heated air pass through the chamber without direct contact with the particles and, therefore, do not contribute directly to the drying process [45]. Differences in feed parameters and interactions occurring inside the spray drying may influence the consistency and quality of the resulting product [42].

Many APIs, such as antibiotics, enzymes, vitamins, and vaccines, can be encapsulated using this technique [45]. The most commonly used materials that build the shell of microcapsules include polysaccharides (natural gums like gum arabic, mucilages, chitosan, cyclodextrins, maltodextrins, modified starch, cellulose, and their derivatives) and proteins (gelatin, sodium caseinate, soy proteins, and whey proteins) [40,41]. Table 2 presents some examples of substances used for coating the microcapsule core by the spray-drying method.

Across the reviewed studies on spray drying, EE showed considerable variability, generally ranging from approximately 50% up to over 90%, depending on formulation composition and process conditions. Polymer selection appeared to be a key factor influencing encapsulation performance within this technique.

### 3.2. Spray Cooling

**Figure 2:**
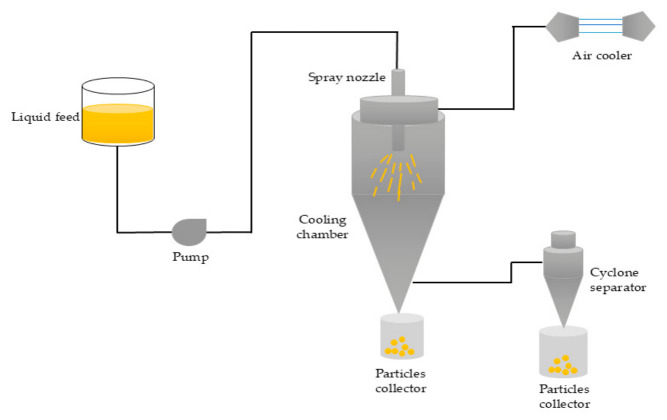
Spray cooling process.

Spray cooling (Figure 2) is a method used to prepare solid microcapsules by atomizing a mixture in which an active ingredient is either dissolved or dispersed in a molten wall material to form lipid droplets, which are then cooled by contact with cold air in a cooling chamber [3,53]. The active ingredient or ingredients is/are incorporated into a lipid matrix, melted lipid material, or an oil-in-water emulsion (in the case of hydrophilic substances) [54]. Unlike spray drying, this process uses cold air flowing through the chamber at a temperature below the melting point of the lipids. The resulting solid particles are collected in a container below the chamber, while the very fine particles are collected in a cyclone separator [54].

This technique enables encapsulation of vitamins, antioxidants, and natural pigments and increases the stability of sensitive compounds, such as small peptides like insulin and drugs with limited solubility [3,54]. Advantages of spray cooling include its scalability, reproducibility, speed, cost-effectiveness, simplicity, precise control over particle size and morphology, ability to increase drug stability, dissolution rate, and protection in adverse conditions, changing their unpleasant taste and flavor, reducing their volatility and hygroscopicity, and use of organic solvents or water [3,53,54,55].

This method also has limitations. The microcapsules obtained in this way can lead to the migration of material from the core of the microcapsule [54]. When choosing the material to be encapsulated, it is crucial to consider substances that are stable at the melting temperature of the lipid matrix [54,55]. Other limitations include fast cooling rates and the low capacity for encapsulating the active ingredients [54].

The most commonly used materials that build the shell of microcapsules include hydrophilic (polyethylene glycol and its esters, and poloxamers) and lipophilic (triglycerides, fatty acids, fatty alcohols, and waxes) matrix materials [56]. In the case of heat-sensitive active ingredients, the appropriate encapsulating materials are lipids with a lower melting point, but these microcapsules have to be stored under controlled temperature conditions [55]. Spray cooling is particularly useful for the preparation of sustained-release formulations and lipid-based drug delivery systems. Table 3 presents some examples of substances encapsulated by the spray cooling technique.

According to the reported studies, spray cooling generally resulted in high encapsulation efficiency, mostly above 80% across different APIs and carrier systems. In some cases, encapsulation efficiency approached or exceeded 100%, particularly in insulin-loaded formulations, indicating efficient incorporation of the active ingredient within the lipid-based matrix.

### 3.3. Fluid Bed Coating

Fluid bed coating is a method that involves the encapsulation of suspended solid core particles in a chamber using a stream of air, while a polymer solution or dispersion (coating material) is sprayed onto them [3,8,12,61]. Particles that are carried by the air flow in this way continuously fall and return to the top of the chamber, which is why their coating with polymer solution is uniform, in several layers. The coated particles are then transferred to a zone where the applied layer hardens by cooling or solvent evaporation [3,12]. These three steps (suspending, spraying, and cooling) are repeated until the desired wall thickness of the microcapsule is achieved [8]. In one study, components of Mexican plum fruit (*Spondias purpurea* L.) extract, including phenolic compounds, anthocyanins, ascorbic acid, dehydroascorbic acid, and total vitamin C (ascorbic acid and dehydroascorbic acid), were microencapsulated using spray drying and spout-fluid bed drying methods [62]. The results showed that microcapsules derived from the spout-fluid bed drying technique have better antioxidant capacity retention, encapsulation efficiency, and protection of these compounds than microcapsules derived from the spray drying process.

**Figure 3:**
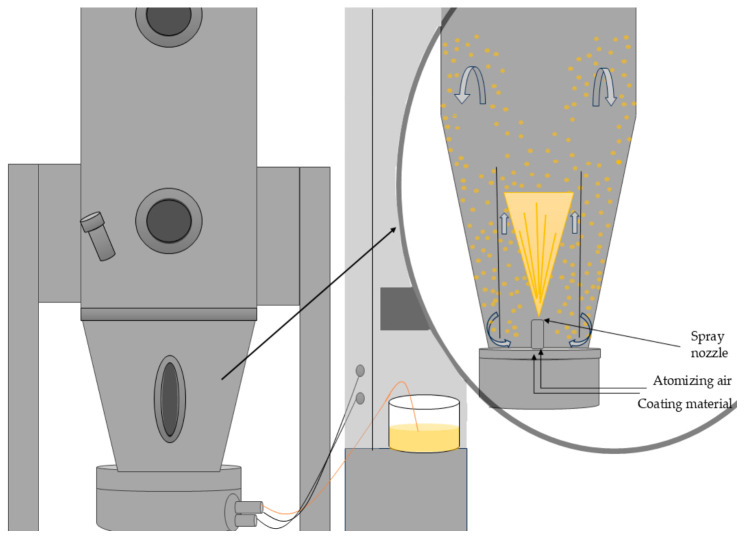
Bottom spray coating process.

There are three types of this method [3]:

- Top spray coating. The polymer solution is sprayed from top to bottom, in the opposite direction to the fluidizing air.
- Bottom spray coating (Figure 3). The polymer solution is sprayed from the bottom upward, in a co-current direction to the fluidizing air. The nozzle is positioned at the bottom of the chamber, spraying the liquid coating material onto the fluidized core particles.
- Tangential spray coating. This method involves the coating of solid particles when they are brought together with the coating material in the gap between a raised tangentially positioned rotating disk and the bottom of the coating chamber.

Advantages of this method include cost-effectiveness, good encapsulation efficiency, and the production of spherical, compact, dense, and uniform capsules with good flow properties [61]. This method is suitable for heat-sensitive substances.

The droplets of the coating material must be smaller than the active substance particles, and the air flow rate has to be controlled, which are limitations of this method [61].

### 3.4. Freeze Drying

Freeze drying or lyophilization is one of the most commonly used methods for microencapsulation of active ingredients in the food and pharmaceutical industry [63]. After preparation of a polymer solution containing the active substance, this mixture is atomized into droplets. The next steps are as follows [3,11,63,64,65,66]:

1. Pre-freezing. The droplets are frozen using liquid nitrogen or a cryogenic freezer to form solid ice crystals.
2. Primary drying (sublimation). Under vacuum, water or another solvent is removed by sublimation, i.e., the ice sublimates from the solid to the gaseous phase, without passing through the liquid phase. Microcapsules with a porous structure are formed.
3. Secondary drying (desorption). The residual water or solvent is removed at higher temperatures than in primary drying.

During primary drying, temperatures of −50 °C to −30 °C and pressures of 0.05 to 0.1 mBar are commonly applied [67]. Because this method uses low temperatures, it is suitable for the encapsulation of heat-sensitive compounds [68]. Lyophilized products can be reconstituted quickly, so they can be used for the encapsulation of vaccines and antibodies when rapid administration is required [69]. Therefore, freeze drying remains one of the most important stabilization approaches for biologics, vaccines, antibodies, and other pharmaceutical products sensitive to heat and moisture. Other advantages include a reduction in oxidation, since the process occurs under high vacuum, and an extended shelf life of the product due to reduced microbiological activity. On the other hand, this method has some disadvantages. It is more complex and costlier, and it requires more time than other drying techniques, because it involves controlling parameters such as freezing rate, vacuum pressure, and temperature [3,67,70]. Freeze drying produces more porous microcapsule structures (due to sublimation) that release active compounds faster, which is why this method provides less protection to the core material [63,65]. If the final product is porous, completely dried, and well soluble, it becomes hygroscopic and, unless it is directly dried and sealed in the final package, it must be stored and packaged under special conditions. Table 4 presents some examples of substances used for coating the microcapsule core by the freeze-drying technique.

According to the reported studies, freeze drying generally provided relatively high encapsulation efficiency, ranging approximately from 78% to nearly 90%, depending on the formulation composition and wall materials used. Compared to spray drying, freeze-dried formulations had more porous structures, which contributed to faster release of encapsulated compounds.

### 3.5. Solvent Evaporation

Solvent evaporation is a method widely used in the pharmaceutical, food, cosmetic, and textile industries. Depending on the hydrophilicity or hydrophobicity of the substance being encapsulated, this method has multiple approaches.

Encapsulation of hydrophobic active substances by solvent evaporation [74]:

- The oil-in-water (o/w) method. This method is used for encapsulation of hydrophobic active substances. The polymer is dissolved in a volatile organic solvent into which the active substance is also dissolved, forming an organic phase that is emulsified in an aqueous phase that contains a stabilizer. Removal of the solvent by extraction and evaporation (by heat or reduced pressure) leads to the solidification of the organic phase droplets into microcapsules.

Encapsulation of hydrophilic active substances by solvent evaporation [74]:

- The water–oil–water (w/o/w) double emulsion method. An aqueous solution of a hydrophilic substance is mixed with an organic phase, a W/O emulsion is formed, and then the resulting emulsion is dispersed into a second aqueous phase.
- The oil-in-water (o/w) dispersion method. The active substance is dispersed in the form of solid particles in a solution of organic solvent and polymer.
- The oil-in-water (o/w) co-solvent method. The addition of co-solvents (methanol, ethanol) improves the solubility of the hydrophilic substance in the organic phase.
- The oil-in-oil (o/o) non-aqueous method. The aqueous phase was replaced by an oil phase.

If the solvent is removed by extraction, more porous microcapsules are formed [8]. Polymers used to build the shell using the solvent evaporation method are polymethyl methacrylate, polylactide, poly(lactide-co-glycolide), ethyl cellulose, and polyhydroxybutyrate [8,74]. The organic solvent used in this encapsulation method should be non-toxic and easily volatile with a low boiling point, with the ability to dissolve the polymer and to be poorly soluble in the external phase. Such solvents are most often methylene chloride, ethyl acetate, or chloroform [74]. One study showed satisfactory results in increased thermal stability and controlled release of capsaicin, using polylactic acid as the polymer, dichloromethane as the solvent, and polyvinyl alcohol as the emulsifier in the oil-in-water (o/w) method [75]. Capsaicin encapsulation is expected to provide benefits like masking its pungent odor. A study investigating propolis extract encapsulation by the double emulsion solvent evaporation method, using ethylcellulose, poly(D,L-lactide-co-glycolide), and polycaprolactone as wall materials, polyvinyl alcohol as the surfactant, and dichloromethane as the organic solvent, reported good antioxidant (especially with ethylcellulose as the wall material) and phenolic (particularly when polycaprolactone was used as the wall material) encapsulation efficiency [76].

Solvent evaporation is a low-cost, reproducible, scalable, and simple method requiring minimal operating skills, used for the microencapsulation of drugs, biomolecules, proteins, and food ingredients, enabling their controlled release and masking unpleasant tastes [3,77]. The disadvantage of this method is that organic solvent residues may remain in the final formulation [77].

### 3.6. Coacervation

Coacervation is the first described method of microencapsulation. This technique is one of the most common methods for preparing microcapsules and finds application in many industries, such as the pharmaceutical industry. It is based on the phase separation process, i.e., the formation of a coacervate, whereby the coacervate (a polymer-rich phase) surrounds the core of the active substance, building the microcapsule shell. It is primarily used for encapsulating hydrophilic substances [5].

Coacervation begins dispersing or dissolving the core material in a polymer solution. By changing conditions, such as pH, temperature, or the addition of salt, a non-solvent, or another polymer, and depending on the type of coacervation (simple or complex), phase separation occurs into two phases: a viscous, polymer-rich phase (the coacervate) in the form of small droplets, and a polymer-depleted phase [5,7,8,11]. The formation and properties of this coacervate phase are governed by intermolecular interactions such as electrostatic attraction, hydrogen bonding, and entropy-driven effects, which determine its internal structure and stability. Recent studies have shown that these physicochemical characteristics of coacervates play a key role in defining the morphology and functional performance of the resulting microcapsules [78]. The coacervate droplets surround the core material, forming the microcapsules. Chemical or enzymatic cross-linkers such as urea, formaldehyde, glutaraldehyde, genipin, or a heat treatment process can stabilize and harden the microcapsule shell. Because of the electrostatic interaction between two aqueous phases, which leads to the transformation of the liquid into a gel, microcapsules are formed [5]. At the end, microcapsules are separated from the solution by filtration or centrifugation, then washed and dried [7,11].

This technique is divided into simple and complex coacervation. In simple coacervation, an active substance (core material) is dispersed in the solution of one polymer. By changing environmental conditions, such as through the addition of salts or water-miscible liquids that are poor solvents for the polymer (e.g., ethanol, acetone, or propanol), the solubility of the polymer decreases, leading to desolvation, phase separation, and the formation of coacervates. Also, coacervation can be caused by changing the pH value and temperature [6].

**Figure 4:**
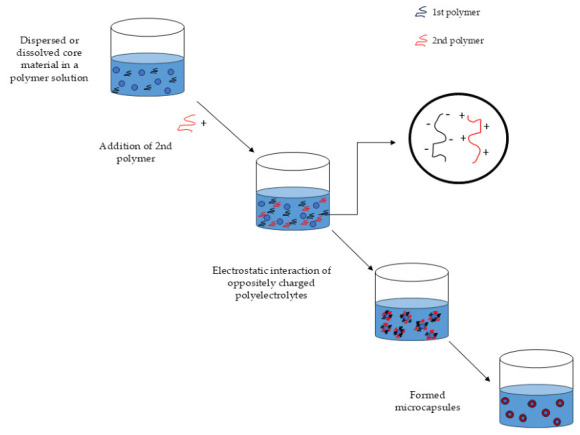
Complex coacervation.

Complex coacervation (Figure 4) involves the reaction between two or more oppositely charged polymers, most often polysaccharides and proteins, where the difference in polymer concentrations must be large enough to allow mutual interaction, but not precipitation [6,79]. The active substance is dispersed in a solution of one polymer, then another polymer of the opposite charge is added, with changes in the environmental conditions. An insoluble complex is formed by the electrostatic interaction of oppositely charged polyelectrolytes, which is deposited at the phase boundary and forms the microcapsule shell. The advantages of using complex coacervation include higher encapsulation efficiency, the possibility of controlled release of the core contents, and the formation of water-insoluble and heat-resistant microcapsules [79]. These properties make complex coacervation particularly attractive for the development of pharmaceutical drug delivery systems requiring controlled release, mucoadhesion, and protection of sensitive active ingredients. Other advantages include the lack of organic solvents and particle size control. However, this method gives the best results only within a certain range of pH values and is additionally influenced by temperature, ionic strength (in the presence of certain electrolytes and colloidal solutions), and protein/polysaccharide ratio [5,80,81,82].

The most commonly used polyelectrolyte pair is gelatin/gum arabic and gelatin/alginate, where gelatin is the polycation and gum arabic or alginate is the polyanion. Other wall materials used for encapsulation by coacervation are chitosan, carrageenan, whey protein isolate, casein, carboxymethylcellulose, and ethyl cellulose. Table 5 presents some examples of substances used for coating the microcapsule core by the coacervation technique.

According to the reported studies, coacervation techniques generally achieved moderate to high encapsulation efficiency, typically ranging from approximately 75% to over 95%, depending on the polymer system and formulation conditions. Among the evaluated techniques, coacervation exhibited the highest encapsulation efficiency values. It can be observed that gelatin, pectin, and chitosan are the most commonly used natural polymers in coacervation studies.

A summary of the main characteristics of the microencapsulation techniques discussed in this section is presented in Table 6, highlighting their main features, including principles, wall and core materials, particle size, encapsulation efficiency, advantages, and limitations.

## 4. Natural Polymers Used as Coating Materials

The selection of an appropriate coating material is one of the most important factors determining the encapsulation efficiency, release profile, and stability of microcapsules. Coating materials should be tasteless, cheap, chemically inert with the core material, capable of protecting the substances from the undesirable environmental conditions, and able to provide stability and strength to the capsules. In addition, the coating material should be non-hygroscopic and water-soluble, which is important for achieving controlled release in aqueous systems [3]. The structural and physicochemical characteristics of natural polymers largely determine the formation, stability, encapsulation efficiency, and drug release profile of microcapsules. Therefore, understanding the relationship between macromolecule structure and pharmaceutical function is essential for the rational design of natural polymer-based drug delivery systems.

**Figure 5:**
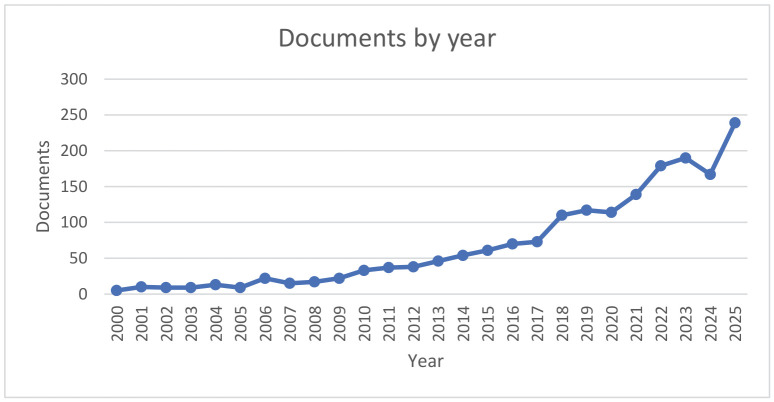
Number of publications related to natural macromolecules published between 2000 and 2025 in the Scopus database.

To illustrate the research trends in the field of natural macromolecules, a bibliometric analysis was conducted using the Scopus database. Publications related to natural macromolecules published between 2000 and 2025 were analyzed, and the annual number of publications is presented in Figure 5. The search results revealed a total of 1798 documents. The number of publications increased over time, from 5 publications in 2000 to 239 publications in 2025, indicating a growing scientific interest in natural macromolecules and their applications.

The subject area analysis indicated that microencapsulation is most commonly reported in the fields of Chemistry, Agricultural and Biological Sciences, and Materials Science. It also has significant contributions from Engineering, Pharmacology, Toxicology, Pharmaceutics, Medicine, Physics, and Astronomy, illustrating its multidisciplinary nature. In addition to the overall publication trend for natural macromolecules, separate analyses were performed for individual natural polymers commonly used in microencapsulation for the period 2000–2025. Based on the data provided, the two most commonly used natural polymers are alginate (258 publications) and chitosan (237 publications), followed by maltodextrin, gum arabic, and gelatin. Other natural polymers, including pectin, agar, and hyaluronic acid, were also reported, although less frequently.

### 4.1. Alginate

Alginate is a linear polysaccharide that contains residues of α-L-guluronic acid (GG blocks), linked by α-1,4-glycosidic bonds, and residues of β-D-mannuronic acid (MM blocks), linked by β-1,4-glycosidic bonds, and MG blocks composed of alternating β-D-mannuronic and α-L-guluronic acid residues [92,93]. It is the most commonly used polymer for microencapsulation of active substances due to its properties: it is biodegradable, biocompatible, mucoadhesive, and non-toxic, with a porous structure, it forms solid, thermostable hydrogels in the presence of divalent cations in an aqueous environment, and it can encapsulate a wide range of active substances (hydrophilic and hydrophobic compounds, thermolabile substances, and viscous oils) [92,93,94,95,96,97,98]. Alginates form hydrogel microcapsules by reacting with divalent ions, primarily Ca^2+^, which bind to carboxyl groups on different polymer chains, creating a three-dimensional network. In this way, a structure resembling an egg-box is formed [92,95]. The design considerations can enhance the performance of drug delivery systems. These combined properties make it suitable for the design of microcapsules with high loading efficiency and controlled release behavior. The properties of alginate microparticles are affected by formulation and processing parameters, including alginate concentration, the type and concentration of cross-linking agent, oil type, emulsifiers, pH control, stirring speed, cross-linking time, and the proportion of mannuronic (M) and guluronic (G) residues [99]. Alginate rich in GG blocks produces biodegradable gels with increased strength and rigidity [95]. The higher affinity of GG blocks for calcium ions strengthens the gel, while MM and MG blocks enhance its flexibility [92,95]. To dissolve alginate in water before microcapsule formation, the solution needs to be heated to 60–80 °C [99].

Formulations generally contain alginate at levels of 1–6% (*w*/*v*) [92]. Alginate microparticles are used for controlled drug delivery and for the preparation of topical formulations, wound dressings, mucoadhesive tablets, and other formulations. Despite these advantages, alginate alone cannot serve as an oral delivery system due to its degradation in the acidic conditions of the stomach [98]. Due to the porosity of the resulting alginate-coated particles, the core contents can be released rapidly [100]. Insulin [101], glutathione [102], resveratrol [96], *Bifidobacterium BB-12* [103], astaxanthin [104], vitamin E [105], vancomycin [106], and L-ascorbic acid [51] are examples of substances that have been encapsulated using alginate.

### 4.2. Chitosan

Chitosan is a linear polysaccharide consisting of D-glucosamine and randomly arranged N-acetyl-D-glucosamine groups linked by β-(1-4) glycosidic bonds [107]. Like alginate, chitosan also forms a hydrogel. Thanks to its biocompatibility, biodegradability, bioadhesive properties, non-toxicity, antimicrobial and antifungal activity, and anticancer properties, chitosan is used in the fabrication of microcapsules [92,108,109]. When selecting chitosan for use as a coating material in controlled-release systems of the active substance, its degree of deacetylation and molecular weight are important parameters. With increasing degree of deacetylation, the water solubility and biological activity of chitosan increase, and the same applies to chitosan of lower molecular weight [92]. Thanks to the presence of free amino groups in its structure, it acts as a polycation capable of interacting with polyanions such as alginate, pectin, xanthan gum, gum arabic, and carrageenan, thereby forming polyelectrolyte complexes. These structural and physicochemical characteristics of chitosan make it suitable for mucoadhesive drug delivery systems, since the positive amino groups react with the functional groups in mucin. Numerous studies have investigated the encapsulation of substances in the alginate–chitosan complex, as this approach enhances stability, reduces the permeability and porosity of alginate microparticles, and allows controlled and targeted release of the active substance. One study investigating the controlled release of tolmetin sodium, a nonsteroidal anti-inflammatory drug encapsulated in agar–chitosan microparticles, showed that increasing the chitosan concentration prolonged the drug’s release and inhibitory effect on rat paw edema [110]. Such systems also enable the efficient encapsulation of probiotic bacteria, such as *Lactobacillus gasseri* and *Bifidobacterium bifidum*, thereby protecting them from bile salts and prolonging their survival in the gastrointestinal tract [111]. Rutin microcapsules coated with chitosan and soy protein isolate, prepared by complex coacervation, exhibited effective protection of the active compound, significant antioxidant activity, and targeted release of rutin in the intestinal fluids under in vitro tests [112].

### 4.3. Pectin

Pectin is a heteropolysaccharide consisting of α-galacturonic acid residues linked by linear 1,4-glycosidic bonds [107,113]. In addition to its ability to enable controlled and targeted delivery of active compounds in the gastrointestinal tract, pectin reduces inflammation in the gastrointestinal tract, acts as a prebiotic, exhibits the ability to remove toxic metals, and possesses antitumor potential, among many other beneficial properties [114]. Like the previous two polymers, pectin possesses the properties of biodegradability and biocompatibility and the ability to form gels. Due to its ability to create stable emulsions at low concentrations, pectin is particularly suitable for spray-dried microencapsulation [115]. Based on the degree of esterification, pectin is categorized as high-methoxyl (above 50%) or low-methoxyl (below 50%) [113,116,117]. Low-methoxyl pectin forms a gel in the presence of divalent cations, like calcium ions, while high-methoxyl pectin forms a gel in acidic conditions [117]. These physicochemical and structural properties enable the formation of stable microcapsules with protection of active compounds during gastrointestinal transit. Due to its biodegradability, pectin has proven to be a suitable carrier for targeted delivery of bioactive compounds to the colon, where they are absorbed [118]. In addition, it is water-soluble, thermally stable, resistant to low pH, and resistant to enzymatic degradation during digestion [119]. The pectin/alginate microcapsules achieved high retention (83%) and release efficiencies (90.35%) when pectin/alginate 3/0.75% was formulated, demonstrated stability at pH 4, and showed potential for protecting phenolic compounds [120].

### 4.4. Agar

Agar is a natural linear polysaccharide obtained from seaweed, specifically from certain species of red algae belonging to the division *Rhodophyta* [121,122]. It is made of agarose and agaropectin. Agarose is a polysaccharide consisting of alternating units of β-D-galactopyranose and 3,6-anhydro-α-L-galactopyranose, while agaropectin has a similar structure but is modified by the presence of sulfate esters, methoxyl groups, and pyruvic acid ketal (4,6-O-(1-carboxyethylidene) substituent) [122]. Agar is widely used in pharmaceutical, cosmetic, and food products: it acts as a prebiotic, is used in formulations for the targeted release of substances in the gastrointestinal tract, exhibits a laxative effect, and increases intestinal peristalsis by swelling and expanding the intestinal volume. It is used as a gelling agent, stabilizer, thickener, and disintegrating agent in tablets [123]. It is used in the formulation of creams, gels, lotions, shampoos, and toothpaste. Like other polysaccharides, it is biodegradable, biocompatible, and non-toxic. Agarose can inhibit bacterial adhesion and growth on silicone rubber surfaces and, therefore, on medical devices [124].

The main component responsible for the gelling properties of agar is agarose. At high temperatures (90–100 °C), agarose dissolves in water as its hydrogen bonds break. Upon cooling (30–40 °C), these bonds reform, creating double helices that cross-link into a three-dimensional, thermoreversible gel [125]. The difference between melting and gelling temperatures of agar is known as hysteresis, which is a significant property responsible for its thermoreversible gelation behavior and broad application in biomedical and pharmaceutical fields, including controlled drug delivery, encapsulation, and hydrogel formation. Thanks to this property of forming hydrogels, it is used to make microcapsule shells. Therefore, this gelation mechanism allows effective encapsulation and protection of bioactive compounds, improving their stability and delivery performance.

Agar is most commonly used in combination with other polysaccharides for the production of microcapsule shells. Thus, when applied together with gelatin in a study on the development of agar–gelatin microcapsules (prepared by the coacervation method) containing berberine hydrochloride and gallic acid, it was shown to be effective and safe for their delivery, both orally and topically. About 70% of the drug was released from microcapsules with berberine chloride after 72 h in an in vitro skin model, and approximately 90% from microcapsules with gallic acid after 12 h in a simulated gastrointestinal model [126].

### 4.5. Gum Arabic

Gum arabic is composed of polysaccharides and glycoproteins. It is used as an emulsifier, emulsion stabilizer, binding agent, viscosity modifier, and wall material for the production of microcapsules and nanoparticles. In addition to biocompatibility, biodegradability, and non-toxicity, gum arabic exhibits good water solubility, low viscosity, and thermal stability, making it a suitable material for microencapsulation [127,128]. In a study that investigated the release, stability, and antioxidant activity of tea essential oil in microcapsules coated with gelatin, gum arabic, and n-butyl cyanoacrylate by coacervation and in situ polymerization, it was proven that encapsulation of the essential oil improves its thermal stability, prolongs the release, and preserves the antioxidant activity over a longer period of time [129]. The electrostatic interaction between the positive charge in gelatin and the negatively charged groups of gum arabic (the addition of acid to the solution of these two components reduces the pH value and thus creates the corresponding charges) allows the formation of a stable polymer complex that helps prepare microcapsules. Almayda, Masruri, and Safitri used gum arabic to coat extracts of *Ruellia tuberosa* and *Tithonia diversifolia* by lyophilization [128]. Encapsulation efficiency was high, 84,29% when gum arabic concentration was 4% (*w*/*v*), at pH 5 and a stirring time of 60 min. A sustained release of active compounds was not pH-dependent. The arabinogalactan glycoprotein in gum arabic gives good emulsifying properties to gum arabic, which can be used in the microencapsulation of active substances [130]. In pharmaceutical formulations, gum arabic is often used as a stabilizing and encapsulating agent for bioactive compounds, particularly in spray-dried microparticles intended for oral administration [36].

### 4.6. Maltodextrin

This encapsulant is a carbohydrate produced by partial hydrolysis of starch, composed of D-glucose units linked mainly by α-(1,4)-glycosidic bonds [131]. Maltodextrin is one of the most widely used substances in the microencapsulation process due to its thermal stability, low cost, good solubility and digestibility, low viscosity, neutral flavor, prebiotic characteristics, antioxidant properties, and ability to improve the solubility of hydrophobic substances [132,133,134,135]. All of these properties make maltodextrin suitable as a carrier in microencapsulation due to its ability to improve the stability of active ingredients. Because of its low emulsification properties, it should be combined with other wall materials to produce stable emulsions and microcapsules [134,136]. Maltodextrin is widely used in pharmaceutical spray drying processes due to its ability to improve powder flowability, enhance the stability of active compounds, and facilitate oral dosage form development [136].

Lavender essential oil was successfully encapsulated using spray drying as an encapsulation technique and gum arabic and maltodextrin as encapsulating agents in order to protect the oil components [137]. Microencapsulation of anthocyanins may protect these compounds from adverse environmental conditions. A study that investigated this issue used different combinations of wall materials for the encapsulation process, among which was maltodextrin. Barberry (*Berberis vulgaris*) extract, a rich source of anthocyanins, was used for spray drying encapsulation with three different wall materials: a combination of maltodextrin and gum arabic, maltodextrin and gelatin, and maltodextrin [138]. Formulation containing maltodextrin and gum arabic as wall materials, with a core/wall ratio of 25%, resulted in the highest encapsulation efficiency and better protection of the anthocyanin compounds among the tested samples [138]. Maltodextrin can also be used to encapsulate garlic oil via the coacervation method with gelatin, helping to protect the essential oil compounds from adverse conditions [139].

### 4.7. Hyaluronic Acid

Hyaluronic acid is a polysaccharide composed of disaccharide units of D-glucuronic acid and N-acetyl-D-glucosamine, which are linked alternately by β-1,4- and β-1,3-glycosidic bonds [140,141]. Its properties make it suitable for drug delivery. Hyaluronic acid is a biodegradable, biocompatible, non-toxic substance naturally found in the human body, characterized by its non-immunogenic, non-inflammatory, and wound-healing properties, and excellent water-binding capacity [140]. Hyaluronic acid hydrogels provide a matrix capable of encapsulating active compounds and enabling their controlled and sustained release under physiological conditions [142]. Hyaluronic acid-based microspheres containing PEG 6000 and/or sodium taurocholate prepared by spray drying demonstrated high loading efficiency (95%) and significantly improved nasal fexofenadine hydrochloride bioavailability (in vivo in rabbits, bioavailability was increased up to about 48%), indicating their potential as effective nasal drug delivery systems [143]. Also, hyaluronic acid is used for microencapsulation of cells [144]. Due to its ability to selectively interact with receptors expressed on the surface of cancer cells, hyaluronic acid has become a widely studied carrier for targeted anticancer drug delivery systems [141].

To form a hydrogel, hyaluronic acid needs to be chemically modified [144]. Chemical modification of hyaluronic acid is commonly achieved through conjugation or cross-linking reactions at its carboxyl, hydroxyl groups, or N-acetyl groups, resulting in derivative forms with enhanced mechanical and chemical stability, solubility in aqueous media, clearance, and stronger receptor interactions than hyaluronic acid [145]. Additionally, hyaluronic acid exhibits low cellular adhesion and is rapidly removed from the body after injection [146].

### 4.8. Gelatin

Gelatin is a natural polymer obtained by the partial hydrolysis of collagen. Type A gelatin is produced by acid hydrolysis, while type B gelatin is produced by the alkaline hydrolysis of collagen [147].

Gelatin has the ability to undergo a thermoreversible transformation between sol and gel. By heating an aqueous gelatin solution to a temperature above approximately 38 °C, the gelatin molecules form a coil [148]. When the gelatin aqueous solution is cooled to a temperature below 38 °C, the viscosity increases, and then the gel and triple helix structure are formed [148].

Gelatin is characterized by biocompatibility, film-forming ability, biodegradability, and non-toxicity. It was one of the first polymers used for microencapsulation of active substances. The hydrophilic nature of gelatin is a challenge during the formation of microcapsules, which is why the resulting structures do not have satisfactory strength and rigidity, and approaches are being taken to improve the physical properties by adding various cross-linking agents [149,150]. The amphoteric nature of gelatin allows it to be combined with polysaccharides such as alginate and chitosan. These properties make gelatin one of the most extensively used natural polymers for the preparation of controlled-release and mucoadhesive drug delivery systems.

### 4.9. Other Natural Macromolecules Used as Wall Materials

Table 7 presents additional examples of macromolecules used in the microencapsulation process, including whey protein, casein, soy protein isolate, and carrageenan.

## 5. Pharmaceutical Applications of Natural Polymer-Based Microcapsules

Microcapsules have been investigated for a wide range of pharmaceutical applications, including oral, topical, transdermal, pulmonary, mucoadhesive, and colon-targeted drug delivery, as well as for the stabilization and delivery of proteins, peptides, probiotics, and vaccines. Microcapsule-based systems can address formulation challenges associated with all four classes of the Biopharmaceutics Classification System (BCS). Their potential roles in overcoming class-specific limitations are summarized in Table 8.

One of the important advantages of microcapsules relates to enhanced dissolution, permeability, and mucoadhesion of the APIs. Since a drug must dissolve in the intestinal tract before being absorbed, drugs with poor water solubility show limited absorption and reduced bioavailability, which is why microencapsulation is an option that helps overcome the solubility and permeation problems of the poorly soluble drugs. By coating APIs with mucoadhesive polymers, the contact time of the APIs with the mucosa is extended; they remain at the site of administration for a long time and are more rapidly absorbed into the systemic circulation. By modifying every aspect of ADME (absorption, distribution, metabolism, and excretion), microcapsules can improve pharmacokinetic performance, control the rate and site of drug release, prolong residence time, and reduce toxicity and the need for frequent drug use.

### 5.1. Oral Drug Delivery

Oral administration of the medicines is the most favorable drug delivery route due to its convenience, simplicity, and high patient acceptance. Numerous physiological and biopharmaceutical challenges associated with oral drug delivery have led to the development of systems for targeted and controlled drug delivery, such as microcapsules. Encapsulated medicines are protected from gastrointestinal degradation and released into the colon in a targeted, controlled manner. There are some examples. Budesonide is a corticosteroid that treats ulcerative colitis. To minimize absorption from the stomach and small intestine, as well as first-pass metabolism, and to target inflamed areas, it was packaged in dextran microcapsules and administered to rats [19]. The colonic delivery system significantly reduced inflammation in the colon of rats with colitis after oral administration, compared to the same drug dose given as an oral suspension. Microencapsulation can develop protein and peptide drug delivery systems for their protection from enzymes, factors in the environment (temperature, light, and pH), and for their prolonged release. Insulin, a polypeptide hormone needed for regulating blood sugar levels, given subcutaneously, can lead to some complications like hypoglycemia and skin changes at the injection site. Additionally, insulin is easily degraded by enzymes and stomach acid and is poorly absorbed when taken orally, which highlights the growing need for advanced systems for its oral delivery [58,101,157,158]. Probiotics are live microorganisms essential for maintaining intestinal flora balance and supporting overall health and immunity. The acidic environment in the gastrointestinal tract reduces the survival of orally administered probiotics, which is why scientists are continuously looking at ways to encapsulate probiotics using spray-drying and freeze-drying methods [159]. Microencapsulated formulations should protect them during storage and passage through the gastrointestinal tract, ensure targeted release in the colon, and enhance their ability to colonize the gut [160]. For example, bacteria from the genus *Lactobacillus* [111,150,152,161], *Bifidobacterium* [103,111], and *Saccharomyces* [161] were encapsulated using natural polymers in order to enhance the survival rates during transit through the stomach. In addition to probiotics, certain antibiotics, such as amoxicillin, do not favor the acidic environment in the gastrointestinal tract, which is why there are tendencies to coat them with a combination of polymers such as chitosan and alginate [162].

Microencapsulation not only has the potential to increase drug bioavailability but may also help protect surrounding tissues from damage. An in vivo study on Wistar rats investigating the microencapsulation of ibuprofen and escin using chitosan and xanthan gum as polymers showed no statistically significant changes in liver and kidney biochemical markers compared with the control group, even after 14 days of oral microparticle administration [163]. Also, histological evaluation of ibuprofen-loaded polyelectrolyte complex-based microparticles showed fewer adverse effects on the liver and kidneys compared to ibuprofen suspension, significantly reducing ibuprofen-induced liver and kidney damage [163].

Another limitation of oral dosage forms is the bitter or unpleasant taste of APIs, which can be a barrier for patients, especially in geriatric and pediatric patients, but also in animals. If reduced bitterness can be achieved in oral formulations, patient acceptability and compliance may be higher. The most common techniques used for microencapsulation of drugs whose taste needs to be masked are spray drying, spray cooling, coacervation-phase separation, solvent evaporation, and fluidized bed coating [20]. The taste of chlorpheniramine maleate [21] and enrofloxacin [164] can be masked by microencapsulation techniques.

In oral delivery systems, natural polymers have significant roles due to their numerous benefits as a result of their biodegradable characteristics, biocompatibility, non-toxicity, low production cost, and gel-forming abilities. Polysaccharides are defined by their stability, hydrophilic properties, and ability to undergo structural alterations, making them suitable for use in drug delivery systems [165]. In oral drug delivery formulations, alginate, chitosan, and dextran are frequently used as polymers. Synthetic polymers, such as Eudragit and ethylcellulose, are commonly used in the preparation of microcapsules for taste masking of drugs [166,167,168,169].

Primary routes of drug liberation from microcapsules include diffusion, dissolution, osmosis, and erosion [2,170]. In diffusion-controlled release, the dissolution fluid permeates the polymeric shell and dissolves the encapsulated drug. The dissolved drug then migrates through the porous structure of the shell and is gradually released into the external environment. As the drug content in the core decreases and the system becomes less saturated, the concentration gradient reduces, resulting in a slower drug release rate, and the release is no longer zero-order [170]. Osmosis involves the movement of water molecules down the concentration gradient (from an environment with a lower concentration of solute to an environment with a higher concentration of solute) through the semipermeable membrane, like a polymer shell. Depending on the concentration, physicochemical properties of the active ingredient, shell permeability, and temperature, drug transport across the shell occurs at different rates. Another release mechanism is swelling-controlled release. When water enters the polymer shell, it causes the polymer to swell, allowing the drug to dissolve and gradually diffuse into the surrounding medium. Polymer properties play a key role in controlling the rate of swelling of the system [170]. In erosion/degradation-controlled release, when a microcapsule comes into contact with an aqueous environment, water first infiltrates the polymer shell, causing it to hydrate. This hydration creates micropores that allow the drug to diffuse and be released [170]. Simultaneously, the polymer matrix begins to erode and degrade, which weakens the shell structure and further promotes drug release. Additionally, drug release can be triggered by environmental stimuli such as changes in pH or the presence of specific enzymes. pH-responsive release is usually achieved using pH-sensitive polymers that change their properties depending on environmental pH, which enables drug release, for example, in colon-targeted systems where pH-responsive polymers are insoluble in acidic conditions, while they dissolve at higher pH values, enabling drug release in the colon. Enzyme-triggered release occurs when specific enzymes, such as proteases and lipases, present in the biological environment degrade the polymer shell, leading to the release of the encapsulated drug.

### 5.2. Topical and Transdermal Delivery

The skin is the largest organ in the body, and its primary role is to protect the body from external factors (microorganisms, UV light, trauma, dust, and pathogens). Topical and transdermal drug administration is simpler and non-invasive compared to oral or parenteral methods. There is no enzymatic degradation of drugs, no first-pass metabolism, and no acidic conditions like in the gastrointestinal tract. The large surface area of the skin enables it to absorb a variety of drugs and ingredients found in cosmetic products. In addition to the positive aspects, the application of drugs through transdermal and topical application has its limitations. As the skin consists of several layers, the barrier it forms is stronger, making it more difficult for substances without the proper properties to penetrate. Local irritation and sensitization may also occur.

Microencapsulation may offer an ideal carrier system for active ingredients, allowing prolonged skin retention and controlled dermal release. In order to achieve controlled release of antifungal drugs to heal skin disease caused by fungi, Chun-Wah Marcus Yuen et al. (2012) prepared chitosan/miconazole nitrate and chitosan/clotrimazole microcapsules [171]. The in vitro drug release study indicated that prepared microcapsules could provide a sustained release of drugs for more than 12 h under various pressures and pHs, but more in chitosan/clotrimazole microcapsules (66.1%) in comparison with chitosan/metronidazole nitrate microcapsules (49.5%) under 5kg of pressure at pH 5.5. 5-fluorouracil, an anticancer drug used topically to treat skin cancers, may be encapsulated using chitosan as a polymer [172]. Sustained release for 72 h in the in vitro skin model can be achieved, as well as enhanced cell growth inhibition of human keratinocytes compared to free 5-fluorouracil. Encapsulation also helps reduce the degradation of unstable ingredients in topical formulations, such as vitamin E [24,88,105], retinol [173], catechins [174], and rosmarinic acid [27], or helps overcome skin irritations, as in benzoyl peroxide [29]. Microencapsulation of such ingredients could enable the potential application of these systems in the protection of sensitive bioactive compounds from oxidation, light, moisture, pH changes, and other environmental factors, improving their stability, bioavailability, and efficacy.

Understanding the importance of using products with SPF helps protect skin from the harmful effects of UV radiation, skin cancer, and premature skin aging. Chemicals, known as UV filters, are added to sunscreen products to absorb, reflect, filter, block, or scatter UV rays, protecting skin from sunburn and skin damage [175]. However, these filters can penetrate the skin and cause photoallergic and phototoxic reactions [175,176]. If filters are enclosed in microcapsules, there is a possibility of reducing potential toxicological risks. Octyl methoxycinnamate and butyl methoxydibenzoylmethane were encapsulated using the sol–gel technique into alginate microcapsules, and in vitro transdermal delivery study showed that the release rate of these ingredients reached a constant level, which is important, as it may reduce toxicity by enabling a controlled and sustained release profile and reduction in the percutaneous absorption of UV filters [176].

### 5.3. Pulmonary Delivery

The large surface area of the lungs, rich vascularization, and the presence of alveoli make the lungs an ideal place for fast and efficient absorption of APIs and a suitable place for drug delivery. This route of administration is important in the treatment of respiratory diseases such as asthma, chronic obstructive pulmonary disease, and lung infections. However, respiratory barriers such as mechanical (mucociliary clearance), enzymatic, and immunological barriers restrict the delivery of substances into the lungs [177]. Current inhalation therapies are associated with several limitations, including rapid drug clearance and a relatively short half-life, and deposition of inhaled particles in different regions of the respiratory tract, all of which together reduce drug bioavailability at the target site. Microcapsules represent an innovative approach to improving pulmonary delivery systems.

Current inhalation formulation methods are associated with several challenges related to precise control over particle characteristics such as size, morphology, and crystallinity [178]. Inhalable microparticles produced by the spray-drying technique can overcome these limitations. As already mentioned, spray drying enables precise control over particle size, size distribution, and particle morphology. In order to improve the performance of inhalation formulations, one approach is to modify powder dispersibility. This can be achieved through spray-drying microcapsule preparation, which allows control over particle size, density, and surface morphology. Improved powder dispersibility is associated with particles of lower density and reduced radius of curvature at the contact point [179]. Microparticles of budesonide [180] and dapsone [181] encapsulated in chitosan using the spray-drying technique achieved a mass median aerodynamic diameter (MMAD) of 3.41–3.70 µm (depending on molecular weight of chitosan) for budesonide, and 4.70 ± 0.43 µm for dapsone. Microparticles with an MMAD of 1-5 µm are suitable for deep lung delivery, while those with an MMAD above 5 µm will deposit in the bronchial region [182]. Both studies showed promising applications of these systems for lung diseases and sustained pulmonary release.

### 5.4. Mucoadhesive Delivery Systems

Mucoadhesion involves contact between the mucosa (in the respiratory, reproductive, urogenital, and gastrointestinal systems) and the surface of a pharmaceutical form that contains a mucoadhesive polymer, which adheres to biological membranes and prolongs contact time with the mucosa. The attraction forces between the biological membrane and mucoadhesive must be stronger than the repulsion forces in order to achieve adhesion [183]. In this way, it is possible to prolong the drug’s action at the absorption site, enable targeted drug delivery, avoid first-pass metabolism, reduce the frequency of administration, and enhance bioavailability and patient compliance. Also, such systems allow for achieving a local or systemic therapeutic effect. They are often used for buccal, oral, nasal, vaginal, and ocular drug delivery.

Polymers found in mucoadhesive formulations contain groups that interact and bind to mucus through chemical interactions of non-covalent forces such as hydrogen, hydrophobic, and electrostatic forces [183,184]. They should be biodegradable, biocompatible, mucoadhesive, non-toxic, safe, and have the ability to form strong non-covalent bonds with biological membranes. In addition, mucoadhesion is influenced by several polymer characteristics, including molecular weight, cross-linking density, surface charge, polymer concentration, and swelling capacity [184]. Examples of such polymers include chitosan, sodium alginate, guar gum, gellan gum, hyaluronic acid, gelatin, pectin, cellulose, starch, tragacanth, and xanthan gum [183,184,185].

Mucoadhesive microcapsules have significant applications in modern controlled drug delivery systems due to their efficiency, safety, and potential for improving therapeutic effects. Great attention is paid to vaginal mucoadhesive formulations. Luliconazole, an antifungal drug used to treat vulvovaginal candidiasis, was entrapped in the spray-dried alginate/gelatin microcapsules to overcome problems of low aqueous solubility and shorter retention on the skin surface [186]. Sustained retention of luliconazole-loaded formulations was observed ex vivo, and with an increase in gelatine concentration, antifungal activity increased. Albertini Beatrice et al. (2009) prepared econazole nitrate mucoadhesive microspheres by spray-cooling technique using Gelucire ^®^ 53/10 as a carrier, and in order to enhance the mucoadhesive ability, several mucoadhesive polymers such as chitosan, sodium carboxymethylcellulose, and poloxamers (Lutrol^®^ F68 and F127) were added [59]. The addition of mucoadhesive polymers increased the mucoadhesive properties of the microspheres; however, only the addition of poloxamers 188 and 407 led to statistically significant differences in mucoadhesive properties from the control formulation (only with econasole nitrate) and Gelucire-based microparticles. This may be due to higher compatibility between Gelucire and poloxamers [59]. Synthetic polymers such as poloxamers are often added to vaginal formulations because of their ability to form a thermoreversible gel when the temperature increases [185]. This advantage may cause prolonged drug release and retention on the mucosal membrane.

Microparticulate delivery systems for the nasal, oral, and buccal administration were also studied. Gavini Elisabetta et al. (2005) prepared spray-dried metoclopramide hydrochloride microspheres using sodium alginate and chitosan hydrochloride alone or in combination [187]. In vitro mucoadhesive test showed that the combination of alginate and chitosan in microparticles achieved high adhesion to the filter paper saturated with mucin, ranging from about 81% (large particle size) to 91% (small particle size). Ex vivo permeation studies indicate that due to the presence of chitosan in alginate–chitosan microspheres, the permeation of metoclopramide hydrochloride through sheep nasal mucosa increases, reaching a value of about 70-80% at the end of the test. Chitosan is often used in preparation of mucoadhesive microparticulate delivery systems. Thanks to the hydroxyl and amino groups that react with mucin through hydrogen bonds, and electrostatic attractive interactions between positively charged amino groups and negatively charged sialic acid in mucus, chitosan has good mucoadhesive properties [184,188]. Granisetron [189] and salbutamol [190] were also incorporated into chitosan microspheres for potential nasal drug delivery. Oral mucoadhesive microcapsules were investigated too. Baclofen, an antispastic drug, was microencapsulated using different polymers like chitosan, carbopol 934P, and hydroxypropylmethylcellulose K4M in order to develop sustained-release mucoadhesive microcapsules [191]. The formulated system demonstrated good mucoadhesive properties and prolonged drug release, which may enhance gastrointestinal residence time and reduce dosing frequency. The addition of carbopol prolonged the release of the drug due to the increased mucoadhesiveness of carbopol, suggesting that the addition of synthetic polymers to the formulation can significantly improve the efficacy of the formulations [191]. Ocular microparticles were investigated by Mauro et al., encapsulating sorafenib tosylate, a therapeutic agent used in the treatment of retinopathies, with chitosan functionalized with L-arginine, using the coacervation technique [192]. The microparticulate system, in vitro, showed strong mucoadhesive ability (time- and concentration-dependent) and higher transcorneal permeation compared to the free drug. Such knowledge can enhance formulations for topical ocular drug delivery, thereby improving drug retention at the application site and facilitating drug passage through the cornea.

### 5.5. Colon-Targeted and Site-Specific Delivery

When an API intended to act in the lower parts of the digestive tract is coated with polymers, it can be protected from premature release in the stomach or small intestine. This will allow the substance to be released at the desired location, in the colon, which is a good place for drug delivery due to its physiological properties: large surface area, high blood perfusion, near-neutral pH, lower concentrations of efflux transporters and cytochrome P450 enzymes, prolonged transition time, and diverse microbial environment [193,194,195]. These properties allow some drugs, such as proteins and peptides, that cannot be efficiently absorbed in the small intestine, to be absorbed into the colon. Colon-targeted drug delivery enables local treatment of diseases such as inflammatory bowel disease (IBD) and colon cancer, protects drugs from stomach acid and enzymatic degradation, reduces systemic side effects and dosing frequency, and bypasses first-pass metabolism.

Variations in pH throughout the large intestine complicate the formulation of systems designed to release APIs into the colon. In the cecum, the initial part of the large intestine, the pH reaches about 6.4 and gradually increases toward the transverse (pH 6.6) and distal colon, reaching values of around 7 [196]. Additionally, pH levels can reach 7.0 to 7.5 in the terminal ileum, potentially leading to premature drug release before reaching the colon, which represents an important limitation of purely pH-dependent colon-targeting systems, particularly in the context of IBD therapy. Polymers should not be soluble in the conditions prevailing in the stomach and small intestine. To make this possible, it is necessary to choose polymers that are acidic, i.e., behave like acids [197]. This property of the polymer will allow it to remain insoluble in the acidic conditions of the stomach and to dissociate and dissolve under the more basic conditions of the intestines, especially in the colon, resulting in drug release. Such pH-responsive natural polymers are alginate, hyaluronic acid, and cellulose derivatives [198]. Thanks to mannuronic and guluronic residues in alginate, alginate possesses carboxyl groups, which are responsible for its acidic properties [92,93]. Chitosan is also a pH-responsive polymer, but due to its amino groups being protonated under acidic conditions, it behaves as a polybase [198]. As a polycation, chitosan can interact with polyanions to form polyelectrolyte complexes, often through a complex coacervation process. Chitosan is known for its antibacterial and anti-ulcer potential, as well as for its mucoadhesive behavior [184,188,198].

The human gut microbiome, especially the colon, contains over 400 species of bacteria and is generally composed of five dominant bacterial phyla, including *Firmicutes*, *Bacteroidetes*, *Proteobacteria*, *Actinobacteria*, *and Verrucomicrobia* [196,199]. In addition to its role in food breakdown, immune function, protection against pathogens, and influence on metabolism, the intestinal flora contributes to the targeted delivery of drugs to the colon. It is possible to create systems that will release the substance at a specific location using bacteria found in the intestines. This is possible because bacteria contain numerous enzymes that can digest components used to coat drugs, such as polysaccharides [200]. This approach may lead to the formulation of such systems that will lead to the improvement of the treatment of local intestinal diseases such as IBD and enable controlled release of drugs in the colon.

IBD includes a group of chronic inflammatory bowel diseases, such as ulcerative colitis or Crohn’s disease, that affect the normal function of the digestive tract. Clinically, these diseases are manifested by intermittent, recurring episodes of inflammation affecting parts of the gastrointestinal system with various symptoms such as pain and diarrhea. Anti-inflammatory drugs and immunosuppressants are used in the treatment of IBD. The main routes of administration for colon-targeted drug delivery systems are oral and rectal administration. Due to the variable conditions in the gastrointestinal tract, conventional therapy is being replaced by modern colon-targeted delivery systems. Microparticles show numerous advantages compared to other systems: reduction in local and systemic toxicity, controlled release, extended residence time, protection from acidic conditions in the stomach, rapid passage through the gastrointestinal tract, and rapid delivery of the drug to the colon [193,195]. Natural polysaccharides used for drug coating, such as chitosan, alginate, and pectin, in addition to properties such as biocompatibility, biodegradability, non-toxicity, and low cost, possess prebiotic properties, thus supporting the development of intestinal microflora [193].

### 5.6. Protein, Peptide, Probiotic, and Vaccine Stabilization

Microencapsulation of proteins, peptides, probiotics, and vaccines leads to protection from degradation, sustained release, enhanced mucosal immune responses, improved stabilization, and reduced need for cold-chain storage [160,201,202,203]. The existence of numerous problems related to proteins, such as their high molecular weight, instability in internal and external conditions, limited stability in the bloodstream, increased toxicity, and side effects when administered in relatively high doses, affects their efficacy and application in pharmaceutical formulations [201]. This happens with insulin. As already mentioned, insulin is degraded at low pH and in the presence of enzymes and, due to its physicochemical properties, has limited absorption. In vitro and in vivo studies of microencapsulated insulin demonstrated potential for application in diabetes therapy, as the microcapsules increased stability, sustained release, improved absorption, and therapeutic efficacy of insulin.

Vaccines represent one of the most important achievements of modern medicine. They use the body’s natural defenses, stimulating the immune system to effectively defend the body against pathogens or prevent the progression of disease. Their microencapsulation may induce cellular, humoral, or secretory immune responses [204]. Rocha Cláudia E.V. et al. (2021) prepared alginate–chitosan microcapsules of gamma-irradiated *Listeria monocytogenes* against listeriosis in a mouse model. This research demonstrated that such systems reduced liver damage, resulted in a 60% survival rate in mice after 14 days of infection, and caused increased levels of interferon-ɣ and IL-10 produced by mouse memory cells [205]. Research by Silva Ana Patrícia C. et al. (2015) showed that mice immunized with alginate-encapsulated live attenuated *Brucella spp*. vaccines were protected against infection and had no lesions in their spleens and livers [206]. The possibility of encapsulating *Mycobacterium bovis BCG* has also been studied with the aim of potentially releasing such microparticles at a specific pH [47]. Polymers used in vaccine microencapsulation should protect the virus from gastric acid, preserve the activity of antigens, increase their penetration into gastric mucosa, and have adjuvant properties. Besides vaccines, there is research indicating the possibility of encapsulating immunoglobulins such as immunoglobulin G in order to prolong their release [207]. Probiotics act as adjuvants when used in conjunction with vaccines; they enhance their ability to induce an immune response and stimulate the production of pro-inflammatory cytokines [208]. The recombinant *Lactobacillus plantarum 25*, which is used to produce BmpB antigen (*Brachyspira* membrane protein B) fused with M cell homing peptide, was encapsulated in alginate/chitosan/alginate microcapsules in order to investigate oral vaccine delivery [208]. Several conclusions were reached: 1. Encapsulated probiotics survived in simulated gastric (pH 2.0) and intestinal conditions (pH 7.2) even in the presence of pepsin and bile salts. 2. Survivability of encapsulated probiotics after 8 weeks of storage at 25 °C was 30%, and 60% at 4 °C. 3. More than 65% of probiotics survived in simulated gastric fluid (pH 2.0) and more than 75% in simulated small intestinal fluid (pH 7.2). 4. Encapsulated probiotics were released in a controlled manner in simulated gastric fluid within 12 h. 5. BmpB-specific IgG and IgA production in mice after 4 weeks of the first immunization was achieved by *Lactobacillus plantarum 25*-M-BmpB-microcpasules [208]. This and all other examples point to the important role achieved by microencapsulation of such microorganisms and molecules in increasing their stability, efficiency, and release.

Collectively, these examples demonstrate that natural polymer-based microcapsules represent versatile pharmaceutical platforms capable of improving drug stability, bioavailability, targeting, and therapeutic efficacy across multiple routes of administration (Table 9).

In Table 9, some examples of microparticles that could have pharmaceutical applications are presented.

### 5.7. Emerging Technologies and Future Perspectives in Natural Polymer-Based Microencapsulation

In order to enable targeted drug release and retention at the site of administration, microcapsule surface properties and mechanical stability should be optimized. Recent techniques for preparing microcapsules, such as electrospray and microfluidics, have emerged. These technologies aim to create microcapsules that are smaller, uniform in size, and possess an increased specific surface area, while allowing precise control over their physicochemical properties [209]. Three-dimensional (3D) printing is a revolutionary manufacturing technique in the pharmaceutical industry that enables the creation of targeted drug delivery systems with controlled release of active substances. This technology enhances absorption of APIs through mucous membranes and promotes the development of personalized therapy, reducing side effects and enhancing therapeutic efficacy and patient adherence [210]. Although it is relatively new in pharmaceutical practice, 3D printing is one of the fastest-growing and most-researched areas of modern pharmaceutical production. Compared to traditional pharmaceutical formulations (tablets, capsules, solutions, emulsions, and others), encapsulation of APIs with polymers, particularly natural ones, creates systems that facilitate the targeted delivery of drugs to specific tissues. This targeted delivery method could greatly benefit the treatment of various acute and chronic diseases, as well as cancers. Proteins and polysaccharides are known for their biocompatibility, biodegradability, non-toxicity, and structural versatility, which makes them promising for medical applications. However, future research in natural polymer-based microencapsulation should focus on improving the mechanical and chemical stability of polymers, reducing their sensitivity to enzymatic degradation and environmental conditions, minimizing batch-to-batch variability, and developing encapsulation processes that preserve polymer integrity while ensuring consistent product performance.

While using microcapsules offers significant advantages, there are challenges to consider when formulating these systems. The scale-up of microencapsulation from laboratory to industrial production is associated with challenges, including maintaining consistent product quality, safety, stability, and performance, as well as optimizing critical process parameters and ensuring reproducibility. In addition, industrial production must adhere to Good Manufacturing Practice (GMP) standards. Since the transition from simple laboratory equipment to more robust industrial equipment involves changes in process design, operating conditions, and raw material requirements during microcapsule preparation, compliance with GMP guidelines represents a significant challenge. The energy consumption, expensive equipment, and costly polymers involved in microcapsule production must be carefully considered to develop a method that enables minimal resource usage. The choice of polymers for formulation of microcapsule shells can be difficult, as the polymers selected must have GRAS status. The use of biodegradable polymers does not necessarily guarantee the biodegradability of the final microcapsules, as encapsulation may modify degradation characteristics [13]. In recent years, there has been growing concern about the environmental hazards posed by microplastics. A large number of household and personal hygiene products contain plastic that can accumulate and break down into smaller particles (smaller than 5 mm) called microplastics. According to European agencies, the European Chemicals Agency (ECHA) and the European Environment Agency (EEA), one of the significant sources of microplastics is microparticulate systems. Emphasis should be placed on avoiding non-degradable and insoluble synthetic polymers and favoring natural polymers that are not chemically modified. To ensure the highest standards of quality, safety, and efficacy, microencapsulated products must adhere to the relevant ICH guidelines. This includes evaluating encapsulation efficiency, particle size distribution, and dissolution profiles, ensuring the safety of the polymer, and confirming stability during storage. Several FDA/EMA-approved microencapsulated products have successfully reached the market: Lupron Depot^®^ [211], Sandostatin LAR^®^ [212], and Risperdal Consta^®^ [213].

## 6. Conclusions

The development of microcapsules has significantly advanced pharmaceutical drug delivery by enabling the protection, controlled release, and site-specific delivery of APIs. This review summarizes the most common microencapsulation techniques, the properties of natural polymers used for microcapsule formulation, and pharmaceutical applications of these systems, highlighting their advantages, limitations, and potential for drug delivery applications. Natural polymer-based microcapsules have emerged as versatile delivery systems capable of improving drug stability, bioavailability, and therapeutic efficacy. There is a growing need for more efficient delivery systems, so new methods for controlled and targeted drug release are being developed. By gradually releasing the substances, their longer-lasting effects and a better therapeutic effect are achieved. The importance of microcapsules as carriers of active substances, but also in preserving and improving the stability and biological availability of active substances, represents an important field of research for the future. Masking the unpleasant tastes and odors of active substances improves their organoleptic properties, making products more acceptable to patients and consumers. The most commonly used techniques for microencapsulation are spray drying, spray cooling, fluid bed coating, freeze drying, solvent evaporation, and coacervation. Each microencapsulation method has its own advantages and limitations. One of the most commonly used methods, spray drying, allows the formation of spherical, uniform particles, is a rapid process, and has a favorable cost–efficiency ratio. However, it requires complex and expensive equipment and the use of high temperatures, which can lead to the degradation and loss of activity of volatile and heat-sensitive compounds. On the other hand, coacervation and freeze drying are more expensive, but suitable for encapsulation of thermolabile substances. The solvent evaporation method uses organic solvents to dissolve active substances and polymers, which is a potential limitation due to the possible toxicity of the solvents. Understanding the advantages and limitations of these methods allows one to choose the most appropriate method for research. Natural polymers in the form of polysaccharides and proteins significantly influence the properties of microcapsules. In addition to their favorable safety profile, natural polymers can provide mucoadhesion, pH-responsive behavior, colon targeting, and sustained drug release, making them particularly attractive excipients for advanced drug delivery systems. Microcapsules made of natural, biocompatible, biodegradable, non-toxic, and cost-effective polymers such as alginate, pectin, chitosan, agar, gum arabic, maltodextrin, hyaluronic acid, and proteins, such as gelatin, soy protein isolate, casein, and whey protein, are of particular importance. While natural polymers offer many benefits, formulating products with them presents a challenge. Weak mechanical and chemical stability, degradation due to enzyme activity, sensitivity to environmental conditions (pH, ion presence, and temperature), and batch-to-batch variability present challenges. The selection of a microencapsulation method can be difficult due to the process conditions used in the formation of microcapsules, which can affect the stability of the polymer. Future research should focus on enhancing the stability of microcapsules by modifying natural polymers, cross-linking them, or combining them with synthetic materials.

Future research should focus on the rational selection of natural polymers and microencapsulation techniques according to the physicochemical properties of APIs and the intended route of administration. The growing body of evidence demonstrating successful applications of natural polymer-based microcapsules in oral, topical, pulmonary, mucoadhesive, colon-targeted, protein, peptide, probiotic, and vaccine delivery systems highlights their considerable potential in the development of next-generation pharmaceutical formulations. Their ability to improve drug stability, bioavailability, targeting, and therapeutic performance will likely ensure an increasingly important role in future drug delivery technologies.

## Figures and Tables

**Table 1:** Techniques of microencapsulation.

Physical Methods	Chemical Methods	Physicochemical Methods
Spray drying	Interfacial polymerization	Coacervation
Freeze drying	In Situ Polymerization	Solvent evaporation
Spray cooling	Molecular inclusion complexation	Ionic gelation
Fluid bed coating	Co-crystallization	Sol–gel process
Pan coating		Supercritical CO_2_ assisted microencapsulation
Co-extrusion		

**Table 2:** Examples of microcapsules prepared using the spray-drying method.

Polymers	Core Material	Conclusion of the Study
Green starch, alginate, and carrageenan	Tea extract (*Camelia sinensis* L.)	Encapsulation efficiency (EE) was highest when the tea extract (TE) was encapsulated with carrageenan (92.72 ± 1.8%) followed by alginate (76.63 ± 1.52%) and green starch (60.25 ± 7.14%). Particle size peaked at 1 μm, 1.5 μm, and 15 μm, respectively, whereas the particle size of microencapsulated gallic acid (MG) peaked at 1.6 μm, 1.3 μm, and 1 μm. The highest loading efficiency was achieved using alginate 84,19%. Microencapsulated TE with green starch exhibited the fastest release while MG with starch exhibited the slowest release, attributed to the lower solubility of starch. UV protection was achieved with retention of antioxidant activity [46].
Dextran	Budesonide	Drug loading values remained within the 45–50% range regardless of the drug-to-polymer ratio or dextran molecular weight (MW). Highest dextran MW and polymer content led to lowest release at 24 h (55.1 ± 2.3%) with sustained release behavior under simulated in vitro colonic conditions. Microencapsulation of budesonide may improve its therapeutic efficacy [19].
Chitosan, N-acetyl-D-glucosamine, and poloxamer 407	*Mycobacterium bovis* BCG	Microparticles with chitosan and N-acetyl-D-glucosamine released live bacteria at pH 3.0, and microparticles with chitosan and poloxamer 407 at pH 8.0. Microparticles enable the release of active ingredient at a certain pH value [47].
Alginate, chitosan, hydroxypropyl β-cyclodextrin, and poloxamer 407	Polymyxin B	The mean particle size was 6.7 ± 1.2 μm for polymyxin B microcapsules with alginate (at high concentration), hydroxypropyl β-cyclodextrin (at high concentration), and poloxamer 407 (at low concentration), and 6.5 ± 0.7 μm for microcapsules with the same ingredients but with low-concentration alginate. The in vitro drug release study demonstrated the sustained release properties of sodium alginate, with only 26 ± 3% release observed at 24 h, compared to 94 ± 3% from the free drug solution at the same time point. Microcapsules with chitosan, hydroxypropyl β-cyclodextrin, poloxamer 407, and polymyxin B exhibited a faster release of polymyxin B due its weaker structural integrity. Enhanced antimicrobial activity of polymyxin B microparticles against *E. coli* and *P. aeruginosa* was achieved [48].
Polyvinyl alcohol	Dronedarone hydrochloride	Microencapsulation efficiency was above 50% for all tested samples. Microcapsules that contain the highest levels of polymer demonstrate a sustained release over a duration of 24 hours [49].
Maltodextrin (MD) and gum arabic (GA)	*Boesenbergia rotunda* extract (rich in flavonoids pinostrobin and pinocembrin)	The formulation with lower MD and higher GA content had the smallest particles (3.11 μm) and the highest EE (78.68%), while the formulation with higher MD content and lower GA content produced the largest particles (10.94 μm) with EE of 76.92%. Both formulations showed good thermal stability. The MD–GA ratio of 1:1 showed the best overall performance, offering high solubility, good encapsulation efficiency, low hygroscopicity, and retention of bioactive compounds [50].
Sodium alginate	Ascorbic acid	EE was 93.48% at the beginning, and 92.55% after 30 days of storage. Microcapsules demonstrated the ability to protect ascorbic acid from oxidation during the 30 day storage period [51].
Maltodextrin and gum arabic	Juniper berry (*Juniperus* *communis* L.) essential oil	EE and particle size ranged from 57.10 to 85.14% and 5.41 to 10.74 μm, respectively. Microcapsules of juniper berry essential oil could be used as pesticides, as they exhibited antibacterial and antifungal activities at oil concentrations 1–5% [52].

**Table 3:** Examples of APIs encapsulated using the spray cooling technique.

Encapsulating Agent	API	Conclusion of the Study
Tristearin, stearic acid, or glyceryl behenate	Salbutamol sulphate	The drug loading was 4.72%, with an EE of 94.4. The volume median diameter (Dv(50)) decreased from 51.7–71.4 µm to 12.7–17.5 µm as the atomization air pressure increased from 4 to 8 bar. High EE and sustained release of salbutamol sulphate with glyceryl behenate microparticles were achieved using spray cooling [57].
Glycerol tripalmitate	Insulin	Insulin EE was 107.7 ± 3.36%, 106.7 ± 1.32%, and 103.5 ± 3.75% for 0.5%, 1%, and 2% loading, respectively. Insulin encapsulated in this way can be released for a minimum of 28 days, with the possibility of sustained release for more than six months [58].
Gelucire^®^ 53/10 (mucoadhesive polymers are added: chitosan, sodium carboxymethylcellulose, and poloxamer)	Econazole nitrate	Most particles were sized between 100 and 355 μm, and for all formulations, the yields were higher than 90%. Spray cooling can be used to produce mucoadhesive microparticles for vaginal delivery of econazole nitrate, enhancing its antifungal activity and adherence to the vaginal mucosa for an extended period of time, as well as to improve the drug availability. The best results showed microcapsules made from poloxamers and Gelucire^®^ 53/10 [59].
Gelucire^®^ 50/13 and Gelucire^®^ 48/16	Three BCS class II APIs (carbamazepine, tolbutamide, and cinnarizine)	For all model APIs, measured drug content matched the theoretical value, giving EE > 80%. Good EE and spherical, smooth, non-aggregated microparticles were obtained. It was concluded that the drug solubility depends on carrier, while the drug release profile was influenced by the API solid state in the microparticles [60].

**Table 4:** Examples of microcapsules prepared using the freeze-drying technique.

Wall Material	Core Material	Conclusion of the Study
Maltodextrin, skim milk, beta-cyclodextrin, chitosan, and carboxymethylcellulose	*Citrus x paradisi* L. peel extract (rich in phenolic compounds such as flavanones naringin and naringenin)	The freeze-dried formulation identified as sample L1 (MD 13%, β-CD 5.8%, CMC 1.2%) exhibited a high solubility rate (61.58%), low moisture content (5.07%), and good EE (78.38%). Microcapsules with a more porous structure were obtained using the freeze-drying technique. This led to faster release and lower EE than spray drying [66].
Maltodextrin, modified corn starch, inulin, gelatin, pectin, tamarind gum beta-cyclodextrin, chitosan, and carboxymethylcellulose	Curcumin	Formulation that included inulin, maltodextrin, and tamarind gum as wall materials showed the highest EE (89.44%). Particles distributed around 100 μm. Freeze-dried microcapsules are better encapsulated (higher EE), while spray-dried microcapsules had smaller particle size, smoother surfaces, and more regular shapes. Both methods enhanced curcumin stability in a carbonated beverage and against heat and acidity [71].
Maltodextrin and gum arabic (single component and their combination)	Hop extract (*Humulus lupulus*, L.)	The highest EE (around 79%) was obtained when maltodextrin was used alone as the encapsulating material. Gum arabic achieved the highest amounts of phenolic compounds and bitter acids after processing, while maltodextrin showed highest EE and protection of hop extracts during storage [72].
Maltodextrin and gum arabic	Phenolic compounds obtained from ciriguela (*Spondias purpurea* L.) peel	EE was high (88.76 ± 0.42), while the average microcapsule diameter was 25.19 µm, which was larger than that of the spray-dried sample. The phenolic compounds, quercetin and kaempferol, were found in the freeze-dried extract, while rutin and myricetin predominated in the spray-dried extract. After digestion, rutin was the main phenolic compound in both methods. Microcapsules obtained by both methods retained a high amount of phenolic compounds after 90 days of storage under storage conditions (7 °C or 25 °C) [73].

**Table 5:** Examples of microcapsules prepared using the coacervation technique.

Wall Material	Core Material	Conclusion of the Study
Gelatin A and pectin	Theophylline	The highest yield of coacervate product of gelatin A and pectin was achieved at a gelatin concentration of 80–90%. Coacervate synthesis depends on the ratio and concentration of polymers, pH, and reaction medium. The optimal ratio of gelatin A and pectin to achieve the maximum yield is 42:8 and pH 3.5. EE was highest (75.23%) with the lowest concentration of cross-linker (glutaraldehyde) and a higher percentage of polymer solutions [83].
Gelatin B and chitosan (coacervation and freeze drying)	Curcuminoids	Microcapsules with a cross-linking agent exhibit higher EE (82.69 ± 0.93%), drug loading (16.21 ± 0.22%), solubility, photoprotection, chemical stability during storage periods, and a longer release time (t50% of 22.75 h), following zero-order kinetics. This can be used for topical products with a gradual release of the substance. Volume mean diameter was 40.498 ± 0.175 µm [84].
Sodium alginate and chitosan	Walnut oil (rich in fatty acids, phenols, squalene, sterols, vitamins)	The EE of walnut oil microcapsules reached 91.18% with a yield of 81.78%, under conditions of a 1:1 sodium alginate-to-chitosan ratio, a 3:2 core-to-wall ratio, a total wall concentration of 1.5%, and pH 4. Particles with small size, higher in vitro gastrointestinal digestibility, oxidation stability, and low hygroscopicity were obtained [85].
Gelatin and carrageenan	*Citrus limon* essential oil from (rich in terpenes and terpenoids)	The higher EE (95%) was achieved when using 1.4 g gelatin, 0.2 g carrageenan, and 0.6 mL oil. Burst release followed by sustained controlled release (57.1% cumulative release at 225 min) was achieved in simulated gastrointestinal conditions. Microencapsulation enables the controlled release of terpen compounds even up to 8 months [86].
Gelatin and five polysaccharides (gum arabic, pectin, cashew tree gum, carboxymethylcellulose, and κ-carrageenan)	Cinnamon extract (rich in proanthocyanidins)	Microcapsules prepared with gelatin/κ-carrageenan and gelatin/cashew tree gum showed higher stability of total phenolic compounds and proanthocyanidins during storage (up to 80% retention). Strong flavor and astringency were masked through gelatin/gum arabic and gelatin/κ carrageenan microencapsulation [87].
Fungal chitosan and gum arabic	α-tocopherol	The EE and drug loading of α-tocopherol were 82.6% and 5.27%, respectively, at a 1:1 (*w*/*w*) wall material-to-active ratio. The particle size was 8.5 ± 2.6 µm (D [50]), and this formulation exhibited the highest positive zeta potential under low pH conditions. The potential of using these materials for encapsulation is demonstrated due to their stability and ease of preparation [88].
Whey protein (WP), flaxseed gum (FG)	Resveratrol (in monodiglyceride fatty acids lipid matrix)	Results showed enhanced EE (≈96–99%), antioxidant activity, and resveratrol stability, as well as slower in vitro release compared to control microcapsules without monodiglyceride fatty acids. Drug loading values varied from 1.43 ± 0.15% (1:0.15 WP/FG ratio) to 1.52 ± 0.12% (1:0 WP/FG ratio). Zeta potential measured at pH 3.0 was dependent on the WP/FG ratio, with charge neutralization at 1:0.05 (≈0 mV) and a shift from positive to negative values with increasing FG content [89].
Gelatin and chitosan	*Angelica sinensis* essential oil (AEO)	Microcapsules with a wall-to-core ratio of 1.50:1, a gelatin–chitosan ratio of 10.38:1, and pH 5.33, showed high EE (82.68%). Mean diameter of microcapsules was 2.26 μm. AEO microencapsulation showed high strong antioxidant activity, stability, and bacteriostatic properties. A rapid release of AEO from microcapsules occurred within the first 120 min under simulated gastrointestinal conditions [90].
Gelatin and pectin	β-ionone	EE and particle size of β-ionone microcapsules were 78.9% and 113 μm, respectively. Increased gelatin content led to stronger gelatin–pectin electrostatic attraction and higher zeta potential, decreased with increasing pH. Microcapsules can maintain good thermal stability above 260 °C and enable sustained release of β-ionone (20.6% released after 30 days at the temperature of 4 °C) [91].

**Table 6:** Summary of the main characteristics of the microencapsulation techniques.

Technique	Principle	Wall Materials/Carriers	Typical Core Material	Reported Particle Size/ EE	Main Advantages	Main Limitations
Spray drying	Atomization of solution, emulsion, or suspension in hot air -> solvent evaporation	Polysaccharides (gum arabic, maltodextrin, alginate), proteins (gelatin, casein)	Drugs, probiotics, vitamins, flavors, essential oils	5–5000 µm (average particle size: 5–150 µm) [5,54], EE (≈50–90%) [46,49,50,51,52]	Active, scalable, wide material compatibility, controllable particle size and morphology, sustained release of encapsulated compounds	Use of high temperatures
Spray cooling	Atomization of a molten carrier containing the active compound followed by solidification upon cooling in cold air	Hydrophilic and lipophilic materials	Drugs, natural pigments, vitamins, antioxidants	20–200 µm [54], EE (≈80–100%) [57,58,60]	Cost-effective, scalable, rapid process, precise control of particle size and morphology	Possible core material migration, requirement for substances stable at the lipid matrix melting temperature
Fluid bed coating	Suspension of core particles in an air stream -> spraying of polymer coating -> layer-by-layer coating -> solidification	Polysaccharides (starch, dextran maltodextrin), lipids [5,61]	Vitamins, antioxidants	20–1500 µm [5]	Cost-effective, suitable for heat-sensitive compounds	Requirement for precise control of air flow rate and droplet size (droplets of the coating material smaller than core particles)
Freeze drying	Pre-freezing of droplets, primary drying by sublimation under vacuum, and secondary drying for removal of residual moisture	Polysaccharides (gum arabic, maltodextrin, chitosan), proteins (gelatin)	Probiotics, vaccines, antibodies, phenols	EE ≈ 78–90% [66,71,72,73]	Low-temperature process, excellent for heat-sensitive compounds	High cost, time-consuming, porous structure
Solvent evaporation	Emulsification of a polymer–drug solution followed by solvent extraction/evaporation, leading to polymer solidification	Synthetic polymers (polymethyl methacrylate, polylactide, poly(lactide-co-glycolide))	Drugs, proteins, essential oils, vitamins, antioxidants	0.5–1000 µm [5]	Cost-effective, reproducible, scalable	Organic solvent residues may remain
Complex coacervation	Phase separation of oppositely charged polymers around droplets	Gelatin-gum arabic, gelatin-pectin, alginate–chitosan	Drugs, vitamins, essential oils	2–1200 µm [5], EE ≈ 75–90% [83,84,85,86,88,89,90,91]	High EE, controlled release, mucoadhesion, protection of sensitive compounds	Process depends on pH, temperature, ionic strength, and protein/polysaccharide ratio

**Table 7:** Examples of microcapsules based on whey protein, casein, soybean protein isolate, and carrageenan.

Wall Material	Core Material	References
Whey protein	Vitamin E	[151]
Whey protein and alginate	*Lactobacillus acidophilus*	[152]
Whey protein, flaxseed gum, and monodiglyceride fatty acids	Resveratrol	[89]
Casein and gum arabic	*Lactiplantibacillus plantarum* A3	[153]
Casein and pectin	*Thymus fontanesii* essential oil	[154]
Soybean protein isolate and chitosan	Rutin	[112]
Soy protein isolate, whey protein concentrate, and potato protein isolate	Fish oil	[155]
Carrageenan, green starch, and alginate	Tea extract (*Camelia sinensis* L.)	[46]
Carrageenan and gelatin	*Citrus limon* essential oil	[86]
Carrageenan and alginate	*Salmonella* phage SL01	[156]

**Table 8:** Role of microcapsules in overcoming formulation challenges of different BCS classes.

BCS Class	Main Formulation Problem	Role of Microcapsules
I	Sustained release	Prolonged exposure
II	Poor solubility	Enhanced dissolution
III	Permeability/stability	Protection and retention
IV	Combined limitations	Multifunctional systems

**Table 9:** Representative pharmaceutical applications of natural polymer-based microcapsules.

Route	Therapeutic Goal	Typical APIs	Preferred Polymers	Preferred Technique	Main Advantage
Oral	Preparation of taste-masked microspheres	Chlorpheniramine maleate (CM)	Alginate, chitosan	Ionotropic gelation	EE was 62.2–94.2%. An increase in chitosan and cation concentrations led to a decrease in the drug release rate. Sustained release at pH 1.2 and 6.8 was achieved. CM can be formulated for oral use with acceptable taste [21].
Oral	Preparation of microparticles to measure insulin bioactivity	Insulin	Alginate	Spray drying	EE was 38.2% ± 9.5%. The mean particle diameter was 2.1 ± 0.3 μm. The microparticle preparation process did not affect the bioactivity of insulin, which was 88% ± 15%, as measured by in vitro tests [101].
Oral/ topical	Conduct an in vitro controlled release study, in vitro antibacterial, and in vivo hepatoprotective study	Berberine hydrochloride, gallic acid	Agar, gelatine	Coacervation	Mean drug loading efficiency and mean particle size were 78.16% and 16.75 μm for berberine HCl microcapsules and 70.28% and 21.98 μm for gallic acid microcapsules. Berberine HCl microcapsules released about 70% of the drug over 72 h in the in vitro skin model, while around 90% of gallic acid was released after 12 h in a simulated digestion condition. Sustained release of APIs, enhanced antibacterial effect of berberine hydrochloride, and hepatoprotective activity of gallic acid were achieved [126].
Oral	Evaluation of probiotic release and microcapsules stability on different pHs	*Lactobacillus casei* ATCC 393	Alginate, gelatin	Extrusion	The microparticles were homogeneously distributed with a size of 1.1 ± 0.2 mm. The swelling behavior of alginate–gelatin microcapsules was influenced by pH and ionic strength, with increasing pH (2.4–8.0) and ionic strength (0.01–1 mol L^−1^) leading to reduced stability and faster disintegration. The results showed that probiotic bacteria could be continuously released from the microcapsules in the gastrointestinal tract, with faster and greater release in simulated intestinal fluids [150].
Oral	Targeted and controlled release of probiotics, survival of microorganisms during storage longer than three months	*Lactobacillus acidophilus* and *Saccharomyces boulardii*	Alginate, chitosan	Freeze drying	After 2 h in simulated gastrointestinal fluids, microcapsules protected more than 80% of the bacterial cells. In simulated intestinal fluids, more than 85% of the microorganisms were released. *S. boulardii* was released in the simulated gastric environment, whereas *L. acidophilus* was released after 2 h in the simulated small intestinal environment [161].
Oral mucoadhesive delivery	Reduction in degradation in acidic conditions, improvement of mucoadhesive properties of microcapsules	Amoxicilin	Alginate, chitosan	Ionotropic gelation	The optimum conditions for microcapsule preparation were 2% (*w*/*v*) alginate, 0.75% (*w*/*v*) chitosan (pH 5.0), and 1.0% (*w*/*v*) calcium chloride. The resulting microcapsules showed EE of 84% and an average size of 840 µm. Release followed non-Fickian kinetics (n = 0.43–0.60) with a diffusion- and erosion-controlled mechanism, best fitted by the Higuchi model (R^2^ = 0.968–0.995). Drug degradation was decreased after encapsulation (in vitro): 20.7% after 2 h, 41.9% after 4 h, and 83.3% after 8 h. High bioadhesive strength was achieved [162].
Topical	Improving topical delivery, retention, and bioavailability in the skin	Ascorbic acid, nicotinamide	Chitosan	Spray drying	Mean particle size was 7.53 ± 3.34 μm, and the zeta potential after addition of APIs decreased to 26.0 ± 5.09 mV. EE of ascorbic acid and nicotinamide was 3.65 ± 0.82% and 6.94 ± 1.65%, respectively. Sustained release (in vitro), and (ex vivo) prolonged contact and retention in epidermis/dermis were achieved [25].
Topical	Preparation of microcapsules and investigation of controlled release under simulated cosmetic conditions	Rosmarinic acid	Chitosan/modified chitosan	Spray drying	Mean diameters of 4.2 µm (chitosan) and 7.7 µm (modified chitosan) were obtained. Slower release was observed for chitosan microparticles in water (≈95% after 2 h) and modified chitosan microparticles in oil (≈75% after 2 h), enabling sustained release depending on the formulation type. Chitosan and modified chitosan could be used for sustained release of rosmarinic acid [27].
Pulmonary delivery	Achieving sustained drug delivery in the lungs	Budesonide	Chitosan	Spray drying	Chitosan molecular weight had no significant effect on particle size or drug loading, which remained around 3–4 μm and 4.8%, respectively. Improved therapeutic effect was observed after 7 days of treatment. Microencapsulation may provide sustained release, prolonged effect, and reduced dosing frequency in lung therapy [180].
Pulmonary delivery	Increasing dapsone bioavailability and reducing side effects	Dapsone	Chitosan	Spray drying	Particle size, aerodynamic diameter, and median mass aerodynamic diameter were 7 μm, 4.57 μm, and 4.7 μm, respectively, indicating that these microcapsules can be adequately deposited in the deepest part of the lung. In vivo toxicity study indicated low toxicity of drug-loaded microcapsules and a protective effect of microencapsulation [181].
Nasal mucoadhesive delivery	Enhancing therapeutic response, residence time, and bypass first-pass metabolism	Granisetron	Chitosan	Emulsification cross-linking method	EE was 72.58–79.2%, and all formulations followed zero-order kinetics (R^2^ = 0.988–0.989). Ex vivo, after 6 hours, 71.18% of the drug had permeated through the sheep nasal mucosa. Stability studies showed that microspheres stored at 25 ± 2 °C and 60 ± 5% relative humidity showed maximum stability. Sustained release was achieved [189].
Nasal mucoadhesive delivery	Prolonging release of salbutamol	Salbutamol	Chitosan	Emulsification cross-linking method	EE of various microspheres formulations was 58.36 ± 2.15% to 80.67 ± 0.58%, with average particle size of 4.49–5.97 µm. Results showed good mucoadhesion (80.37 ± 2.37 to 84.46 ± 2.55) and, compared to the oral formulation (12.38 ± 0.57 ng/mL at 3 h), nasal microcapsules showed a similar plasma concentration (12.01 ± 0.46 ng/mL) but with a prolonged release profile (4 h) [190].

## Data Availability

Not applicable.
